# The Effect of Tobacco Smoke *N*-Nitrosamines, NNK and NDEA, and Nicotine, on DNA Mismatch Repair Mechanism and miRNA Markers, in Hypopharyngeal Squamous Cell Carcinoma: An In Vivo Model and Clinical Evidence

**DOI:** 10.3390/curroncol29080437

**Published:** 2022-08-04

**Authors:** Sotirios G. Doukas, Dimitra P. Vageli, Panagiotis G. Doukas, Dragana Nikitovic, Aristidis Tsatsakis, Benjamin L. Judson

**Affiliations:** 1 The Yale Larynx Laboratory, Department of Surgery, Yale School of Medicine, New Haven, CT 06510, USA; 2 Department of Forensic Sciences and Laboratory of Toxicology, Medical School, University of Crete, 71003 Heraklion, Greece; 3 Department of Medicine, Rutgers/Saint Peter’s University Hospital, New Brunswick, NJ 08901, USA; 4 Department of Histology & Embryology, Medical School, University of Crete, 71003 Heraklion, Greece

**Keywords:** DNA mismatch repair, tobacco smoke, *N*-Nitrosamines, NNK, NDEA, nicotine, MSH2, MLH1, human hypopharyngeal squamous cell carcinoma, head and neck cancer

## Abstract

**Highlights:**

- Deregulation in MMR mechanism and miR-21, miR-155 and miR-451a are caused by chronic exposure to *N*-Nitrosamines, NNK and NDEA, and nicotine.
- Reduced *hMSH2* and *hMLH1* gene expression profiles and miRNA deregulations can be detected both at early stages of tobacco smoke-related carcinogenesis and in invasive cancers of the upper aerodigestive tract in smokers.
- MMR gene expression phenotypes are strongly linked to deregulated specific miRNAs, such as miR-21, miR-155, miR-34a and miR-451a.
- The activation of NF-κB may play a role in tobacco smoke-induced MMR dysfunction and carcinogenesis in the upper aerodigestive tract.

**Abstract:**

Deregulation of the DNA mismatch repair (MMR) mechanism has been linked to poor prognosis of upper aerodigestive tract cancers. Our recent in vitro data have provided evidence of crosstalk between deregulated miRNAs and MMR genes, caused by tobacco smoke (TS) *N*-Nitrosamines, 4-(methylnitrosamino)-1-(3-pyridyl)-1-butanone (NNK), in hypopharyngeal cells. Here, we explored whether chronic exposure to TS components can affect MMR mechanism and miRNA profiles in hypopharyngeal mucosa. Using a mouse model (C57Bl/6J wild type) of in vivo 14-week exposure to NNK (0.2 mmol/L) and *N*-Nitrosodiethylamine (NDEA; 0.004 mmol/L), with or without nicotine (0.02 μmol/L), we provide direct evidence that TS components can promote dysplasia, significant downregulation of *Msh2* and *Mlh1* genes and deregulation of miR-21, miR-155, miR-34a, and miR-451a. By analyzing eight human specimens from tobacco smokers and eight controls, we provide clinical evidence of a significant reduction in *hMSH2* and *hMLH1* mRNAs in hypopharyngeal squamous cell carcinoma (HSCC). In summary, deregulation of the MMR mechanism and miRNAs is caused by chronic exposure to TS-related *N*-Nitrosamines, with or without nicotine, in the early stages of upper aerodigestive tract carcinogenesis, and can also be detected in human HSCC. Thus, we encourage future studies to further elucidate a possible in vivo dose-dependent effect of individual or combined *N*-Nitrosamines, NNK and/or NDEA, and nicotine, on the MMR mechanism and their clinical testing to elaborate prognosis and risk assessment.

## 1. Introduction

Tobacco smoking remains a leading risk factor for devastating diseases, such as cancer. Despite the extensive anti-smoking campaign, the prevalence of smokers in the United States and in Europe is estimated to be 14% and 28%, respectively [1,2]. Both active and passive smokers expose themselves to multiple tobacco smoke (TS) carcinogens that increase the risk for malignancies in the upper aerodigestive tract, such as oral, pharyngeal, laryngeal, and hypopharyngeal cancer [3], as well as esophageal cancer [4]. Hypopharyngeal cancer remains one of the cancers with the highest mortality, with a 5-year survival rate that does not exceed 32% [5]. The development of effective methods for molecular analysis has led to the identification of multiple molecular markers with diagnostic and prognostic values [6]. However, the exact way that risk factors such as tobacco smoking promote underlying molecular changes leading to cancer development and progression in the upper aerodigestive tract requires further investigation, which could reveal new biomarkers for the early diagnosis and prognosis of these malignancies

Multiple components in TS have been found to have carcinogenic potency. *N*-Nitrosamines exist in considerable amounts in TS. *N*-Nitrosamines [4-(N-Methyl-N-Nitrosamino)-1-(3-pyridyl)-1-butanone (NNK) and *N*-Nitrosodiethylamine (NDEA) have been shown in vivo to promote carcinogenesis in multiple organs, including the upper aerodigestive tract [7]. Most of the *N*-Nitrosamines found in TS are further metabolized into byproducts with high carcinogenic potency [8]. In parallel to directly affecting the DNA, NNK seems to induce activation of inflammatory cancer-related pathways, such as NFκB and AKT, promoting anti-apoptosis, cell survival and proliferation [9,10]. Our recent in vivo findings provided direct evidence that TS components, such as *N*-Nitrosamines NNK and NDEA, can cause premalignant lesions in the upper aerodigestive tract, accompanied by a significant activation of NFκB and overexpression of an oncogenic mRNA phenotype [6]. Nicotine, another tobacco smoking component, in addition to the well-known addictive effect, seems to promote anti-apoptosis by activating several signaling pathways [11].

Identifying and correcting DNA by several enzyme complexes, such as mismatch repair (MMR) enzymes, is vital for the maintenance of genetic information. Epithelium may often be affected by various harmful agents during its regeneration, causing DNA mismatches. When MMR mechanism is functional, MSH2, which is the main component of the MMR mechanism, forms complexes (such as MutSα) with other MMR enzymes and translocates to the nucleus to recognize base–base mismatches and small nucleotide insertion/deletion. Then, MLH1, with other MMR enzymes, forms complexes, such as MutLα, to repair the identified damage [12]. In the case of a defective MMR mechanism, mismatches remain unrepaired and thus the accumulation of genetic errors increases the risk for tumorigenesis [13]. Congenital or epigenetic defects in mismatch repair mechanisms have been identified as risk factors for multiple malignancies, including those of the upper aerodigestive tract [14].

MicroRNAs (miRNAs) are small non-coding RNAs that have been shown to regulate multiple cellular processes, such as cell proliferation and differentiation [15]. Recent studies have shown that deregulation of miRNAs, which directly control the transcription of oncogenes and tumor suppressor genes, may be responsible for the development and progression of TS-related malignancies [16]. We also recently showed, using an in vitro model, that exposure of hypopharyngeal cells to *N*-Nitrosamines and NNK, at either low or high doses, can lead to deregulation of MMR, likely due to a miRNA dysregulation [17]. However, the in vivo effect of TS components on the MMR mechanism and cancer-related miRNA markers remains unexplored.

Here, we have used our previously established murine animal model to explore whether the chronic exposure of hypopharyngeal mucosa (HM) to TS-related *N*-nitrosamines, NNK and NDEA, with or without nicotine, is capable of decreasing *MSH2* and *MLH1* gene expression as well as deregulating the expression of miRNA markers that were previously linked to upper aerodigestive tract carcinogenesis such as the “oncomirs” *miR-21, and miR-155* and “tumor suppressor*” miR-34a* and *miR-451a* [18,19,20,21,22,23]. In order to investigate the clinical significance of these molecular changes, we also analyzed MMR gene expression profiles in human hypopharyngeal squamous cell carcinoma (HSCC), which had previously demonstrated miR-21, miR-155, miR-34a, and miR-451a deregulations, compared to the adjacent normal tissue (ANT). Our data provide new insights into the molecular mechanism by which TS components promote cancer development and progression.

## 2. Materials and Methods

### 2.1. Animal Model

Our in vivo model has previously been described [6]. Specifically, wild mice C57Bl6J (*Mus musculus*) were treated for 14 weeks with TS components, *N*-Nitrosamines, NNK and NDEA, with or without nicotine (Table 1). Forty 4-wk-old wt-mice (C57BL6J) (20 female and 20 male) were randomly divided into 3 experimental and 2 control groups (*n* = 8 animals in each group). The mice were obtained from Jackson Laboratory (Jax^®^ mice, USA). After acclimatization of mice (2–3 days), treatment exposure, experimental, and their corresponding control procedures were performed in parallel, as described in our approved protocol (11039, by IACUC; Yale University). According to previous studies, the concentrations of *N*-Nitrosamines in TS mainstream, is up to 2950 ng/cigarette for NNK, up to 28 ng/cigarette for NDEA, and 0.5 to 1.6 mg/cigarette for nicotine [24,25,26]. Therefore, it is considered that a 20 pack year smoker can be exposed to approximately 5 mg/kg NNK, 0.05 mg/kg NDEA, and 0.1–0.4 mg/kg nicotine in total [27].

According to these data and previous in vivo models [6,28,29,30] we performed long term exposure to TS components, as follows: (i) nicotine (NIC) (0.02 μmol/L) (Sigma-Aldrich^®^, St. Louis, MO, USA), (ii) *N*-Nitrosamines, NNK (0.2 mmol/L; Santa Cruz Biotechnology, Dallas, TX, USA) and NDEA (0.004 mmol/L) (Santa-Cruz^®^), and (iii) combination of *N*-Nitrosamines with nicotine (NNK-NDEA-NIC), in 2% saccharin in water, which was the sole drinking fluid of the animals for 5 days/week for 14 weeks (Table 1). Saccharin was added to treat the bitter taste of nicotine.

The control groups were as follows: (i) Treated control: 2% saccharin in water was the sole drinking fluid of animals for 5 days/week for 14 weeks, (ii) Untreated control: a group of untreated animals (14 weeks old) was used as negative control (Table 1).

After completion of the experimental procedures, mice were euthanized, according to the latest IACUC policy (by carbon dioxide) and kept on ice until tissue harvest. Four tissue fragments from hypopharynx (HTF) of 2 females and 2 males from each experimental and control group underwent fixation by 10% neutral buffered formalin (Thermo Fisher Scientific, Middletown, VA, USA). Subsequently, fixed tissue samples were embedded in paraffin blocks (Yale Pathology Facilities), for histologic evaluation and IHC analysis. Four more HTF from the remaining mice (2 females and 2 males) from each group were put into a RNA stabilization solution (RNAlater^®^, Life Technologies, Grand Island, NY, USA) and kept in −80 °C, for RNA and miRNA isolation. Total RNA was isolated from representative murine HTF of experimental and control groups, using a RNeasy mini kit (Qiagen^®^, Louisville, KY, USA), according to the manufacturer’s instructions.

### 2.2. Histopathologic Evaluation

HTF from the experimental and control groups were submitted for histopathological evaluation. Four µm thick tissue sections from formalin-fixed and paraffin-embedded HM were stained with hematoxylin and eosin (H&E). Histologic evaluation was performed using light microscopy, based on previously established criteria (WHO, Ljubljana, Gale) [31,32] and laboratory mouse histology [33], as previously described [19,34,35,36]. At least 2 tissue sections from each specimen (8 tissue sections per group; 4 tissue sections per gender per group) were microscopically examined. Normal HM was characterized by stratified keratinizing squamous epithelium with a single layer of basal cells, and was considered as control. In the case of mild grade dysplastic epithelium, architectural disorder and hyperchromatic or pleiomorphic basal cells were topically expanded into the stratum spinosum. Moderate grade dysplasia was characterized by full-thickness nuclear hyperchromatism with a high degree of basal layer expansion and/or nuclear hyperchromatism with an increase of nuclear to cytoplasm ratios, and loss of cell polarity into the middle third of the mucosa. Severe dysplasia or in situ carcinoma was identified by the progression of maturation from base to luminal surface, with atypical cells expanding to the upper layers of mucosa, according to previously assessed criteria [19,31,32,33,34,35,36]. Images were captured and analyzed by Aperio CS2, Image Scope software (Leica microsystems, Buffalo Grove, IL, USA).

### 2.3. Immunohistochemical Staining (IHC)

IHC analysis was performed for MMR proteins. Serial sections (4–5 µM) of formalin-fixed and paraffin-embedded tissues from the experimental and control groups were stained for MSH2 MLH1, and p-NF-κB, following standard protocols.

#### 2.3.1. Chromogenic Staining

In order to observe MSH2 expression and its localization in hypopharyngeal tissue sections, we selected at least 2 tissue sections from each HTF sample (8 tissue sections/group; 4 tissue sections/gender/group). We included tissue sections from HTFs that showed histopathological changes. All sections were stained for MSH2 using 1:100 of anti-MSH2 (D-6) HRP (mouse monoclonal, Santa Cruz Biotechnology., Inc., Heidelberg, Germany). Subsequent tissue sections from each HTF sample were stained for phospho-NF-κB by 1:100 of anti-p-NF-κB p65 antibody (27.Ser 536; mouse monoclonal, Santa Cruz Biotechnology, Inc.). We used 1:100 of anti-mouse IgG secondary antibody conjugated to horseradish peroxidase (HRP;m-IgG1 BP-HRP; Santa Cruz Biotechnology, Inc.) and peroxidase substrate (3–3′ diaminobenzidine tetra-chloride; Santa Cruz Biotechnology, Inc.). In our analysis we used positive controls (human tonsil for NF-κB; MSH2 positive control slides from Cell Marque^TM^ tissue diagnostics), and non-template negative controls, according to the manufacturer’s instructions. Microscopic examination of slides was performed using a Leica light microscope and images were captured using Aperio CS2. Image Scope software (Leica microsystems, Buffalo Grove, IL, USA), which generates algorithm(s), was used to analyze the image. MSH2 “positivity” was assigned as positive nuclei/total number of cells for each section (scores were estimated from 5 images/tissue section; at least 2 tissue sections per group; by Image Scope software).

#### 2.3.2. Immunofluorescence (IF) Staining

We selected at least 2 tissue specimens from each experimental and control group (8 tissue sections per group; 4 tissue sections per gender per group), including specimens with histopathological lesions, to perform IF staining for MLH1, in serial HTF of the selected specimens, using DyLight^®^488 for green (Vector Labs, Burlingame, CA, USA). We used 1:50 anti-MLH1 (A-8) (mouse monoclonal, Santa Cruz Biotechnology., Inc., Heidelberg, Germany). DAPI (blue) (Life Technologies, Thermo Scientific, Waltham, MA, USA) was used to distinguish the nuclei. At the end of IF staining, the slides were examined microscopically and their images were captured for analysis (Zeiss fluorescence microscope, AxionVision system; Carl Zeiss microscopy, White Plains, NY, USA). Total MLH1 expression levels in experimentally treated and control HM were identified by fluorescence intensity (mean ± SD bin count) from 2 independent images from each section [Zen imaging software 2012 blue edition; Carl Zeiss microscopy GmbH; Göttingen, Germany].

### 2.4. Human Specimens

To explore MMR gene alterations in human HSCC from former tobacco smokers, we examined *hMSH2* and *hMLH1* mRNA levels. We enrolled 8 individuals diagnosed with HSCC, comprising 5 men and 3 women (mean age 64 (SD ± 8) years) who were treated by the Yale New Haven Hospital Head and Neck Service. All subjects were former smokers with an average of 37 pack year smoking history (Supplementary Table S1). All tissue specimens were collected prior to chemoradiation therapy and retrieved from the archives of the Department of Pathology (Yale Pathology Tissue Services) according to our Human Investigation Committee approved protocol (HIC#1206010419). The specimens were formalin-fixed and paraffin-embedded (FFPE). Histologic evaluation for tumors and their ANT were previously confirmed by the department of Pathology (Yale New Haven Hospital). Total RNA was isolated from two 10 μm sections from FFPE HSCC and ANTs specimens. Briefly, after sectioning, the tissue was immediately immersed in xylene and RNA isolation was performed using a RNeasy FFPE Kit (Qiagen^®^, Louisville, KY, USA), according to the manufacturer’s instructions.

### 2.5. Quantitative Real-Time PCR (qPCR) Analysis

In order to quantify the mRNA expression levels of MMR genes under long term exposure of HM to TS carcinogens, we performed real-time qPCR analysis. After total RNA isolation, RNA quality and concentration were determined by absorption ratios at 260/280 nm (>2.0) and 260 nm, respectively (NanoDrop^TM^ 1000 spectrophotometer; Thermo Scientific). Subsequently, we performed reverse transcription to cDNA using a Whole Transcriptome kit (Qiagen^®^, Louisville, KY, USA) and real-time qPCR analysis, using specific primers for target and reference genes for mouse (*Msh2*, *Mlh1*, *Gapdh)* or human (*hMSH2*, *hMLH1*, *hGAPDH)* (QuantiTect^®^ primers assay, Qiagen^®^, Louisville, KY, USA) (Supplementary Table S2), and iQ^TM^ SYBR^®^ Green Supermix (Bio-Rad, Hercules, CA, USA), following the manufacturer’s instructions (each sample was assayed in triplicate). We used a Bio-Rad real-time thermal cycler CFX96^TM^ and PCR data was analyzed by CFX96^TM^ software (Bio-Rad, Hercules, CA, USA).

### 2.6. MicroRNA Analysis

MicroRNA analysis was performed to determine the levels of miRNA markers previously linked to MMR deregulations under TS carcinogens or head and neck cancer [17,37,38]. miScript II (Qiagen^®^, Louisville, KY, USA) was used for miRNA synthesis from total RNA, isolated from HM specimens, as described above. Mouse-specific miRNA primers were used to quantify the “oncomirs” miR-21, miR-155, and “tumor suppressor” miR-34a and miR-451a, and RNU6B snRNA as a reference control (miScript Primer Assays, & miScript SYBR Green PCR kit; Qiagen^®^, Louisville, KY, USA) (Supplementary Table S3). Each sample was assayed in triplicate and the data were analyzed using CFX96^TM^ software.

### 2.7. Statistical Analysis

Statistical analysis was performed using GraphPad Prism 7.0 software and one-way ANOVA (Friedman; Dunn’s multiple analysis test; *p* values < 0.05) as well as *t*-test analysis (multiple comparisons by Holm-Sidak) to reveal any evidence of statistically significant reductions of protein, mRNA, or miRNA expression levels in experimental versus control groups. Pearson correlation was performed to estimate the correlation coefficient between expression levels of the analyzed genes and miRNA markers in the studied groups (*p* values < 0.05).

## 3. Results

### 3.1. Chronic Exposure to TS Components Decreases MMR Protein Levels and Promotes Dysplasia in Exposed Hypopharyngeal Mucosa

Histologic evaluation by H&E staining revealed that chronic exposure of HM to TS-related *N*-Nitrosamines, NNK-NDEA, or their combination with nicotine, caused histopathological features of precancerous lesions, such as moderate to severe dysplasia, compared to controls, in line with our prior observations (6) (Supplementary Figure S1). Specifically, HM exposed to *N*-Nitrosamines, NNK-NDEA or NNK-NDEA-NIC, produced moderate to severe dysplastic HM, characterized by architectural changes and hyperchromatic or pleiomorphic basal cells extending into the upper layers of mucosa. On the contrary, controls (2% saccharin in drinking water or untreated HM) demonstrated stratified keratinizing squamous epithelium with a single layer of basal cells, which was characterized as normal HM (Supplementary Figure S1). HM exposed to nicotine alone demonstrated mild dysplastic epithelium, characterized by nuclear hyperchromatic of basal cells and rete ridges (Supplementary Figure S1). Supplementary Table S4 depicts the percentage of C57Bl/6J mice that survived the treatment and the prevalence of mice that developed premalignant lesions under the effect of individual TS components or their combination.

**Figure 1:**
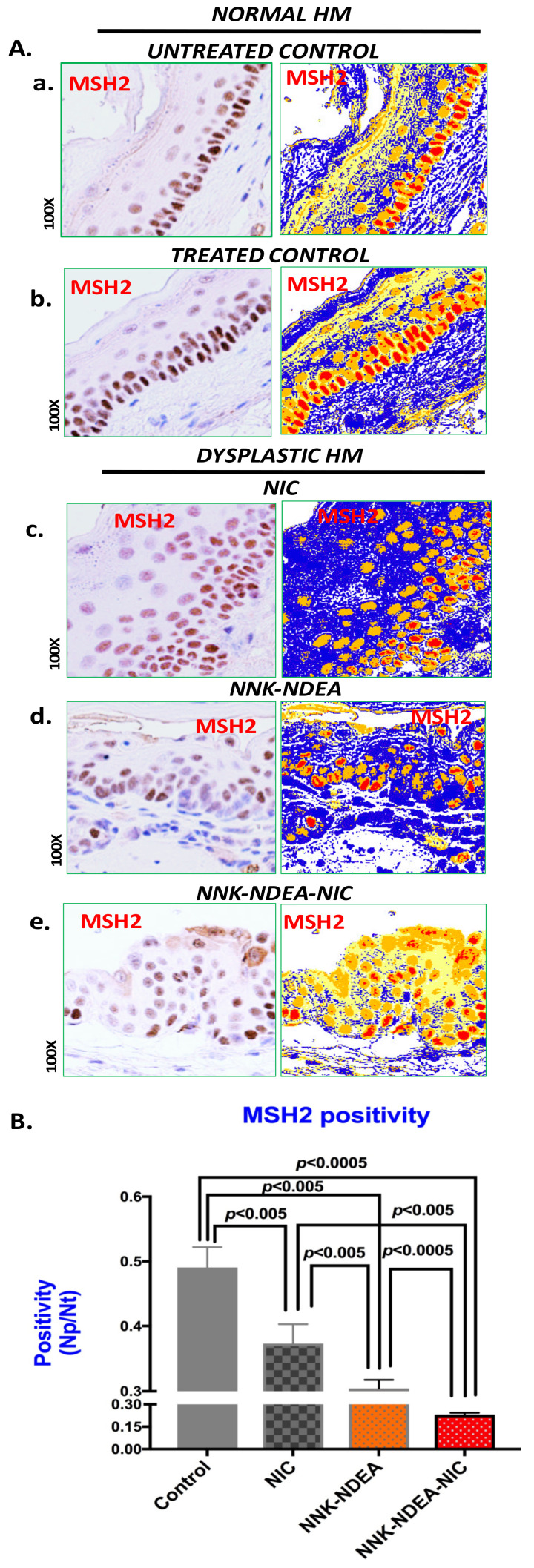
Chronic exposure to TS components decreases MSH2 protein levels and promotes dysplasia in murine HM. (**A**). IHC analysis for MSH2 (brown staining) and image analysis algorithm(s) (red: strong positive nuclear staining; orange: intense positive cytoplasmic staining; yellow: weak cytoplasmic staining; blue: negative staining; by Image Scope software) of hypopharyngeal mucosa (HM) of C57Bl6J mice after 14 weeks of exposure to TS components nicotine (NIC), *N*-Nitrosamines (NNK-NDEA), or their combination (NNK-NDEA-NIC) and controls. (**a**) Untreated control, (**b**) Treated control (normal HM), (**c**) NIC-treated HM (mild dysplastic), (**d**) NNK-NDEA-treated HM (moderate dysplastic), (**e**) NNK-NDEA-NIC-treated HM (severe dysplastic) (original magnification 100×). (**B**). Graph depicts MSH2 nuclear positivity in murine HM-exposed TS components, compared with treated controls, by *t*-test with multiple comparisons using the Holm-Sidak method (*p* < 0.05). (MSH2 nuclear positivity indicates the number of positive nuclei/number of total nuclei).

IHC analysis for MSH2 revealed that, similarly to untreated controls (Figure 1A(a)), normal control treated-HM (exposed to 2% saccharin alone) presented an intense positive nuclear and cytoplasmic MSH2 staining of basal/parabasal cells (Figure 1A(b)). On the contrary, dysplastic HM exposed to nicotine (Figure 1A(c)), NNK-NDEA (Figure 1A(d)) and, in particular, to NNK-NDEA with nicotine (Figure 1A(e)) presented sporadic nuclear or cytoplasmic MSH2 staining in few basal/parabasal or suprabasal cells.

Scoring of nuclear MSH2 revealed that HM exposed to TS carcinogens produced significantly lower levels of MSH2, compared to controls (*p* < 0.05, *t* test; mean ± SD; multiple comparisons by Holm-Sidak) (Figure 1B). Interestingly, HM exposed to NNK-NDEA with nicotine demonstrated the lowest levels of MSH2 positivity (*p* < 0.05, *t* test) (Figure 1B) (Table 2).

**Figure 2:**
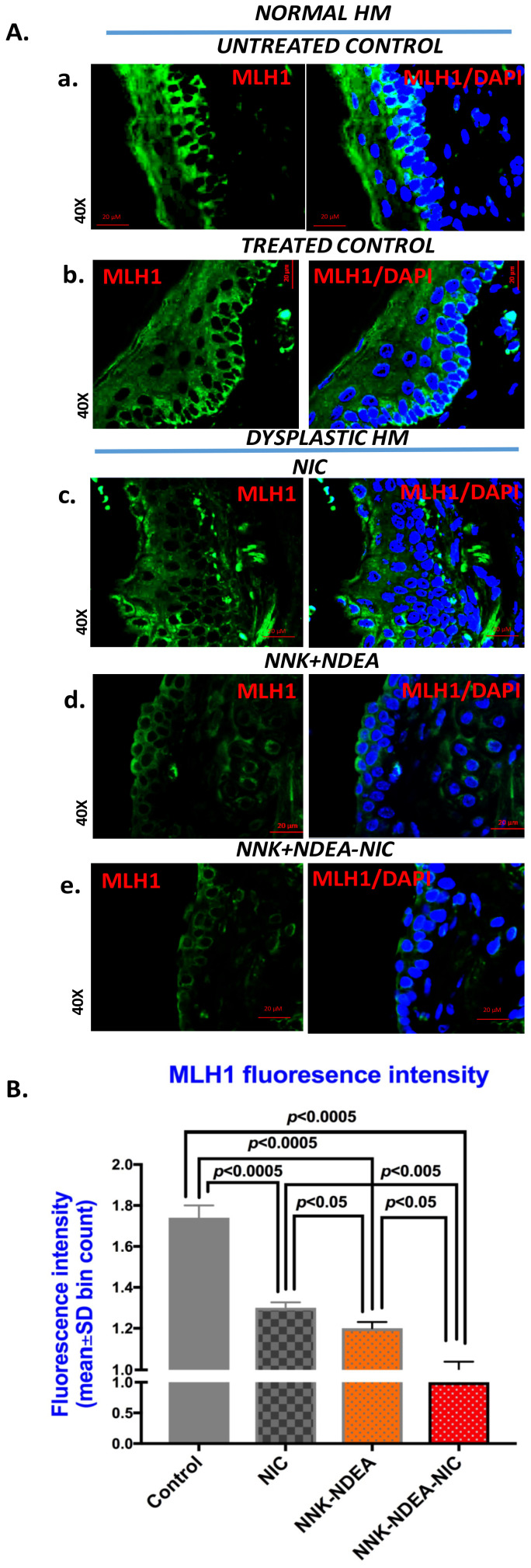
Chronic exposure to TS components decreases MLH1 protein levels in dysplastic murine HM. (**A**). Immunofluorescence staining for MLH1 (DyLight^®^488 for green; blue: DAPI for nuclear DNA staining; scale bar 20 μm by Zen imagining software). (**a**) Untreated control, (**b**) Treated control (normal HM): intense cytoplasmic staining in basal/parabasal cells, (**c**) NIC-treated HM (mild dysplastic), (**d**) NNK-NDEA-treated HM (moderate dysplastic), (**e**) NNK-NDEA-NIC-treated HM (severe dysplastic) (original magnification 40x). (**B**). Graphs created by GraphPad Prism 7.0, demonstrate statistically significant differences of scores (mean ± SD) for MLH1 staining between TS components treated HM vs. controls (*p* < 0.05 by *t* test; multiple comparisons by Holm-Sidak; GraphPad Prism 7.0). (Scores of fluorescence intensity were derived from two independent images (>10 cells per image) from each section (at least two tissue sections per group), using Zen imaging software).

Immunofluorescence analysis for MLH1 revealed similar results to MSH2. Normal treated control or untreated HM showed intense cytoplasmic staining for MLH1 of basal/parabasal cells, similarly to untreated controls (Figure 2A(a,b)). On the other hand, dysplastic HM exposed to nicotine alone (Figure 2A(c)) produced weak MLH1 staining through all thicknesses. Additionally, HM exposed to NNK-NDEA (Figure 2A(d)) or its combination with nicotine produced a weak cytoplasmic MLH1 staining of its dysplastic epithelium (Figure 2A(e)).

Scoring of nuclear MLH1 revealed that HM exposed to NNK-NDEA, with or without nicotine, produced significantly lower levels of MLH1, compared to controls (*p* < 0.05, *t* test; mean ± SD; multiple comparisons by Holm-Sidak) (Figure 2B) (Table 2).

### 3.2. Chronic Exposure to TS Components Downregulates MMR Gene Expression and Deregulates Cancer-Related miRNA Markers in Exposed Hypopharyngeal Mucosa

**Figure 3:**
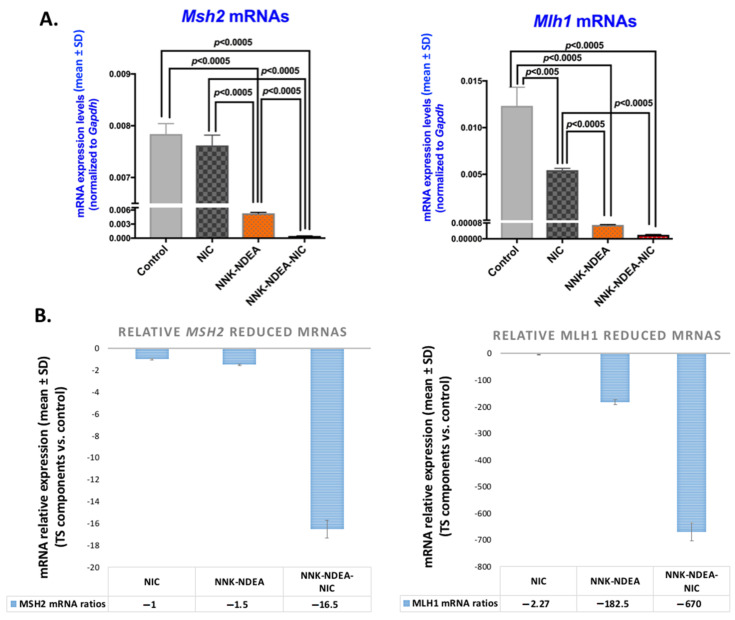
Downregulation of MMR genes in murine HM exposed to TS components. (**A**). Graphs depict mRNA levels of MMR genes, Msh2 and Mlh1 (mRNA levels of genes normalized to Gapdh; by real-time qPCR analysis) in 14-week exposed HM to TS components, nicotine (NIC), *N*-Nitrosamines (NNK-NDEA), or their combination (NNK-NDEA-NIC), and treated controls. (*p* < 0.05 by *t* test; multiple comparisons by Holm-Sidak; GraphPad Prism 7.0; data obtained from four analyzed samples). **(B**)**.** Graphs depict the mRNA ratios of MMR genes, Msh2 and Mlh1, in HM exposed to smoke components relative to controls (normalized mRNAs (mean ± SD) to *Gapdh* reference control).

Gene expression analysis for MMR genes revealed that chronic exposure to TS components induced a significant downregulation of *Msh2* and *Mlh1*, compared to treated controls (Figure 3A; Supplementary Table S5A) (*p* < 0.0005 by *t*-test; GraphPad Prism 7.0). In particular, the combination of NNK-NDEA with nicotine induced the most intense reduction of MMR genes in exposed HM (Figure 3B; Supplementary Table S5A).

**Figure 4:**
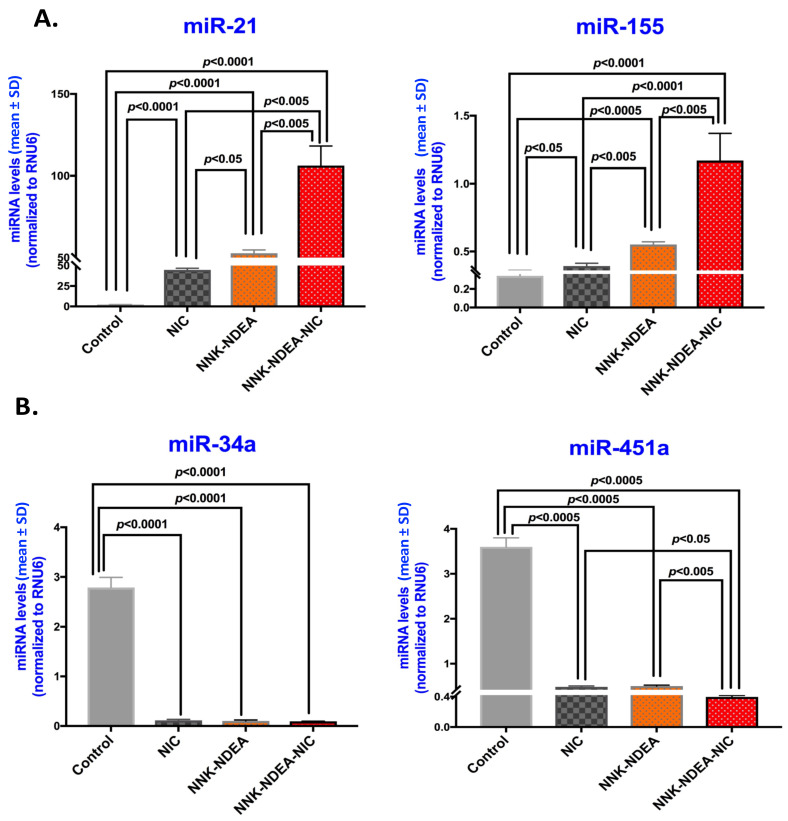
Deregulation of cancer-related miRNA markers in murine HM exposed to TS components. Columns of graphs created by GraphPad Prism software 7.0 show expression levels for (**A**) “oncomirs” miR-21 and miR-155 and (**B**) “tumor suppressor” miR-375 and miR-451a, in 14-week exposed HM to TS components, nicotine (NIC), *N*-Nitrosamines (NNK-NDEA), or their combination (NNK-NDEA-NIC), and treated controls. (miRNA levels (mean ± SD) of each marker were normalized to RNU6, by real-time qPCR analysis) (*p* < 0.05 by *t* test; multiple comparisons by Holm-Sidak; GraphPad Prism 7.0; data obtained from four analyzed samples).

MicroRNA analysis demonstrated that in vivo exposure of HM to TS components induced a significant upregulation of the “oncomirs” miR-21 and miR-155, compared to controls (*p* < 0.0005) (Figure 4A; Supplementary Table S6), as was previously demonstrated in vitro [17]. The combination of NNK-NDEA with nicotine induced the highest miR-21 and miR-155 expression levels. Additionally, chronic exposure to either nicotine itself, NNK-NDEA, or their combination induced a significant downregulation of “tumor suppressor” miR-34a and miR-451a, compared to treated controls (*p* < 0.0005) (Figure 4B; Supplementary Table S6).

Pearson analysis revealed a significantly inverse correlation between (i) miR-21 and MSH2 protein levels (*r* = −9856, *p* = 0.0072) or *Msh2* mRNA levels (*r* = −0.9234, *p* = 0.0383; one-tailed), and (ii) *Msh2* mRNAs and *miR-155* (*r* = −0.9935, *p* = 0.0065; two-tailed), as well as a significant linear correlation between *Mlh1* mRNAs and miR-34a (*r* = 0.9027, *p* = −0.0487) or miR-451a (*r* = 0.9043, *p* = 0.478; two-tailed).

### 3.3. TS Components Induce Activation of NF-κB That Correlates with MSH2 and miRNA Expression in Hypopharyngeal Mucosa

The IHC analysis revealed that NNK-NDEA, with or without nicotine, induced a significant activation of NF-κB, compared to treated controls (Supplementary Figure S2A), in line to our previous findings [6]. Specifically, our data showed that, although nicotine alone could not promote a strong NF-κB activation, its combination with *N*-Nitrosamines accelerated NF-κB activation, particularly in dysplastic lesions (Supplementary Figure S2B).

Pearson correlation revealed a significant inverse correlation between mRNA or protein expression levels of MSH2 and p-NF-κB (*r* = −0.9616, *p* = 0.0192, and *r* = −0.93, *p* = 0.0338) and between p-NF-κB and miR-21 (*r* = −9, *p* < 0.05) levels, in murine HM exposed to TS components.

### 3.4. Downregulation of hMSH2 and hMLH1 Genes in Human HSCC Tumor Specimens from Tobacco Smokers

**Figure 5:**
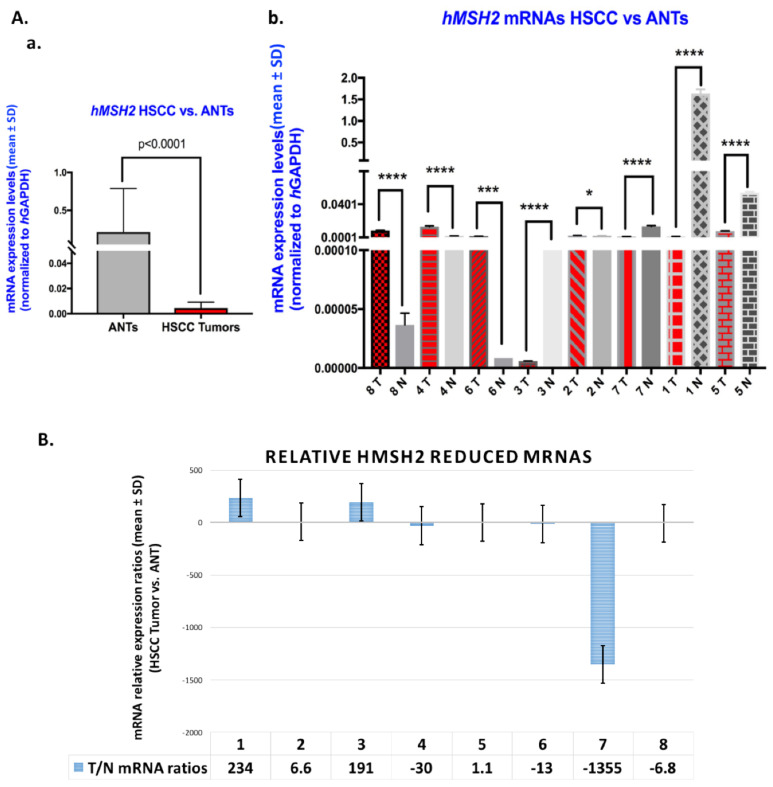
Downregulation of the *hMSH2* gene in human HSCC from tobacco smokers. (**A**). Graphs depict mRNA levels of *hMSH2* (mean ± SD) were normalized to *hGAPDH;* by real-time qPCR analysis) for (**a**) total and (**b**) each of the analyzed human HSCC excised from tobacco smokers compared to corresponding adjacent normal tissue (ANTs). (by *t* test; *, *p* < 0.05; ***, *p* < 0.0005; ****, *p* < 0.00005; multiple comparisons by Holm-Sidak; GraphPad Prism 7.0; data obtained from at least three repetitions). (**B**). Graph depicts the mRNA ratios of *hMSH2* gene in HSCC from smokers compared to ANTs (normalized mRNAs to *hGAPDH* reference control).

**Figure 6:**
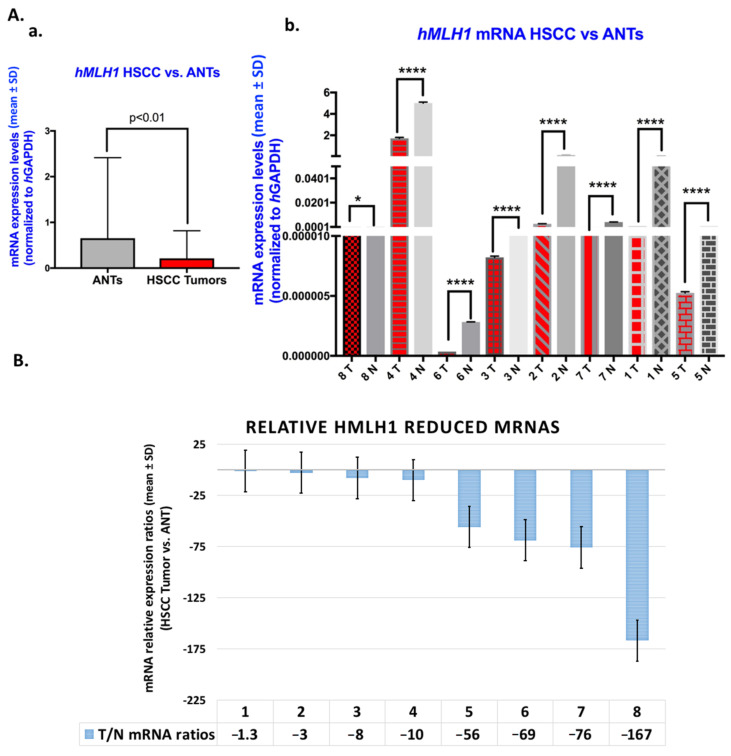
Downregulation of *hMLH1* gene in human HSCC from tobacco smokers (**A**). Graphs depict mRNA levels of *hMLH1* (mean ± SD) were normalized to *hGAPDH;* by real-time qPCR analysis) for (**a**) total and (**b**) each of the analyzed human HSCC excised from tobacco smokers compared to corresponding adjacent normal tissue (ANTs). (by *t* test; *, *p* < 0.05; ****, *p* < 0.00005; multiple comparisons by Holm-Sidak; GraphPad Prism 7.0; data obtained from at least three repetitions). (**B**). Graph depicts the mRNA ratios of *hMLH1* gene in HSCC from smokers compared to ANTs (normalized mRNAs to *hGAPDH* reference control).

Gene expression analysis for MMR genes in human HSCC from former tobacco smokers confirmed significant downregulation of the MMR genes, *hMSH2* (Figure 5A(a,b),B) and *hMLH1* (Figure 6A(a,b),B), compared to their ANTs (*p* < 0.0005 by *t*-test; GraphPad Prism 7.0) (Table 3; Supplementary Table S5B). In particular, *hMLH1* was downregulated in all analyzed HSCC, compared with their ANT (Figure 6B; Supplementary Table S7). Table 3 presents the human MMR mRNA phenotype, as well as miR-21, miR-155, and miR-451a, phenotypes, that we had previously identified in these specimens (Table 3; Supplementary Table S7) [37].

**Figure 7:**
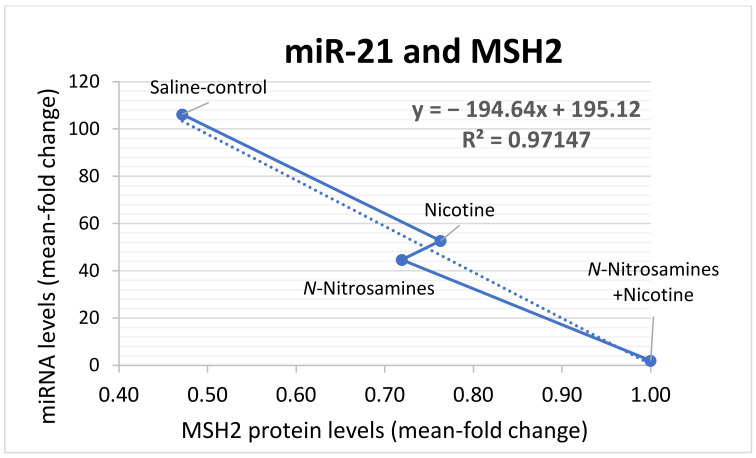
The impact of miR-21 deregulation on MSH2 protein output caused by TS components. Total MSH2 protein levels reduced by the upregulation of “oncomir” miR-21 expression in tobacco smoke components-exposed hypopharyngeal mucosa. (*r* = −9856, *p* = 0.0072; by Pearson analysis). (MSH2 protein levels: mean of total MSH2 immunostaining/total number of cells quantified by Image scope; miR-21 levels: mean of expression levels normalized to *RNU6* by qPCR).

Pearson analysis revealed a significant inverse correlation between expression changes of *hMSH2* mRNAs and miR-21 (*r* = −0.9856, *p* = 0.0072; one-tailed; 95% confidence interval) (Figure 7) and between expression changes of *hMSH2* mRNAs and miR-451a (*r* = −0.8979, *p* = 0.0025; two-tailed; 95% confidence interval).

## 4. Discussion

**Figure 8:**
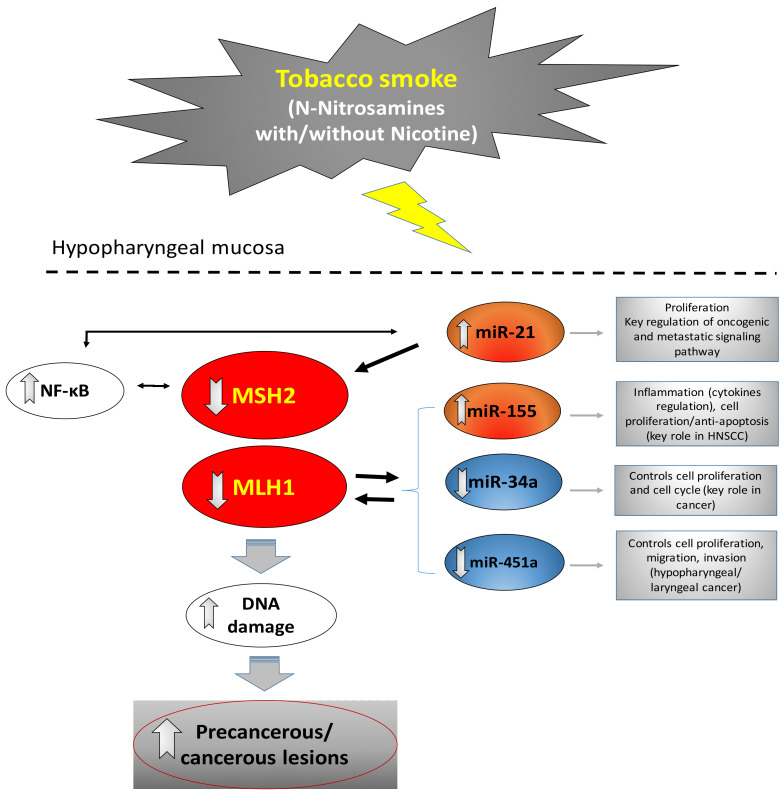
The in vivo chronic effect of tobacco smoke components, *N*-Nitrosamines, NNK and NDEA, and nicotine, induced dysregulated MMR mechanism and miRNA deregulation in carcinogenesis of the upper aerodigestive tract. MMR gene expression phenotypes are strongly linked to deregulated specific miRNAs, such as miR-21, miR-155, miR-34a and miR-451a. The activation of NF-κB may also play a role in tobacco smoke-induced MMR dysfunction, DNA damage and carcinogenesis in the upper aerodigestive tract, interacting with miR-21 and MSH2.

Tobacco smoking is a known risk factor for carcinogenesis of the upper aerodigestive tract [39]. TS components, such as *N*-Nitrosamines, are potential carcinogens [28,40]. We recently showed, using an in vitro study, that exposure of hypopharyngeal or lung cancer cells to *N*-Nitrosamines, like NNK, at either low or high dose, can similarly lead to deregulation of MMR genes likely due to miRNA dysregulation [17]. Based on these in vitro findings, we concluded that NNK, even at a low dose, can contribute to a dysregulated MMR mechanism and increased cell survival rates, indicating a non-dose-dependent effect of TS-related *N*-Nitrosamines. We have also recently shown that the combination of *N*-Nitrosamines, NNK and NDEA, at concentrations based on values previously measured in the mainstream of TS [24,25,26] and used in in vivo models [6,28,29,30], could promote pre-neoplastic lesions in in vivo exposed murine hypopharyngeal mucosa, possibly through the activation of NF-*κ*B and its related oncogenic pathways [6]. On the other hand, we have shown that the combination of tobacco smoke *N*-Nitrosamines with other risk factors, such as bile reflux, was particularly noxious, as it could accelerate the carcinogenic process by causing invasive cancer [6]. Here, we used an in vivo murine experimental approach involving a long time treatment (14 weeks) of the hypopharynx to TS-related *N*-Nitrosamines, NNK and NDEA, with or without nicotine, investigating for the first time whether chronic exposure to TS components can affect the MMR mechanism and miRNA markers, previously linked to carcinogenesis of the upper aerodigestive tract [19,35,36,41]. We used a mixture of NNK and NDEA, with or without nicotine, at concentrations based on our prior in vivo model and other studies [6,24,25,26,28,29,30]. Our novel in vivo experimental data provides direct evidence that chronic exposure of the murine hypopharyngeal epithelium to TS components can induce a defective MMR mechanism and deregulation of cancer-related regulatory miRNA molecules in premalignant murine HM, providing new insights into the mechanism of TS-related carcinogenesis of the upper aerodigestive tract (Figure 8). Our current study revealed significant pathological changes after 14 weeks of treatment, especially in the groups treated with *N*-Nitrosamines and nicotine, encouraging us to extensively investigate the effect of individual *N*-Nitrosamines, NNK and NDEA, and their combination with nicotine at variable concentrations and multiple time points of this process. This revealed the step-by-step progression to carcinogenesis, as well as possible gender-related differences. In order to provide clinical evidence, we also analyzed MMR mRNA phenotypes in human HSCC specimens from former smokers and their ANTs, which previously demonstrated miRNA deregulations [37]. Similarly to our in vivo data, we provide clinical evidence of MMR gene downregulation, particularly of *hMLH1*, in HSCC tumors from former chronic smokers, compared to their ANTs.

Findings from our in vivo model documented that *N*-Nitrosamines, particularly in combination with nicotine, can suppress MSH2 and MLH1 levels in exposed HM (Figure 8). These observations were demonstrated by a decrease in nuclear MSH2 and total MLH1 protein levels, as well as their mRNA levels, in the NNK-NDEA-exposed premalignant HM, compared to normal control, suggesting a dysfunction of MMR mechanism during tumorigenesis. Our current in vivo findings are in line with our previous in vitro data, showing that direct exposure to TS-related NNK can lead to lower expression of MSH2 and MLH1 [17]. As was shown before, a decrease in nuclear MHS2 often indicates a defective or dysregulated MMR mechanism and an elevated risk for malignant transformation [42]. Although mismatch repair genes are expected to be upregulated as a response to a harmful stimulus, such as NNK, downregulation signifies a considerable risk for the accumulation of mutations.

Previous studies have proposed a decrease in MSH2 and MLH1 levels in head and neck malignancies [43]. Although the underlying mechanism requires further exploration, several studies have supported the epigenetic downregulation of mismatch repair genes as a complex process in which specific miRNAs may play a key role [44,45,46,47,48,49,50,51]. Here we showed that chronic exposure of murine HM to TS components led to significantly elevated levels of the “*oncomirs*” miR-21 and miR-155 and a decrease in “*tumor suppressors*” miR34a*,* and miR-451a (Figure 8), in line with our previous in vitro model [17]. In addition, a statistically significant inverse correlation was noted between miR-21 and MSH2 proteins (Figure 7) and mRNA levels, suggesting a possible interaction between “oncomir” miR-21 and MSH2 (Figure 8) (19). Both miR-21 and miR-155 have been suggested to be involved in MMR regulation [17,45,46,51]. Specifically, we recently showed that NNK-induced downregulation in MSH2 expression can be prevented by inhibiting miR-21 [17], while, other studies have reported similar findings, suggesting a miR-21/MSH2 regulatory axis [45,46]. Based on our current in vivo and previous in vitro findings, not only miR-21 but also miR-155 may be directly involved in the regulation of MSH2 gene expression and therefore play a role in MMR function [51].

The tumor suppressor miR-451a has been suggested to be involved in head and neck carcinogenesis [52]. The tumor suppressor miR-34a is also a well-known marker for smoking-associated malignancies, especially head and neck cancer, with a critical role in the regulation of apoptosis and cell proliferation [53,54]. Here we showed for the first time a significant downregulation of miR-451a by TS-related *N*-Nitrosamines or nicotine and the possible interaction with MLH1 (Figure 8). Our data also demonstrate a significant downregulation of miR-34a in NNK-exposed hypopharyngeal mucosa and reveal a significant correlation between miR-34a and MLH1 expression (Figure 8). These data further support the implication of tumor suppressor miR-451a and miR-34a in TS-related carcinogenesis, suggesting further investigation of their important involvement in regulating the MMR mechanism.

Our data also showed that exposure of murine HM to tobacco smoking components, such as NNK-NDEA with nicotine, can lead to moderate to severe dysplasia, in line with previous observations in vivo exposed HM to NNK-NDEA [6,17], supporting *N*-Nitrosamines as promoters of carcinogenesis. According to our recent findings, NF-κB may play a key role in carcinogenesis of the upper aerodigestive tract related to TS *N*-Nitrosamines, by activating other anti-apoptotic pathways [6]. Here, we confirm these data and further document that TS-related *N*-Nitrosamines, with or without nicotine, can endorse NF-κB activation, particularly at sites of dysplastic HM (Figure 8) (Supplementary Figure S2). Moreover, based on our observations, the significant negative correlation between activated NF-κB and MSH2 or miR-21 may further support the role of NF-κB in a TS-induced mutator phenotype, which may lead to a precancerous/cancerous epithelium of the upper aerodigestive tract (Figure 8).

Although the carcinogenic potency of nicotine is still debated, there is evidence that it promotes cell proliferation by activating multiple signaling pathways [11]. Here, we show that not nicotine but *N*-nitrosamines, alone or in combination with nicotine, can cause more severe dysplastic changes and more significant dysregulated MMR mechanism and “oncomirs” miR-21 and miR-155 deregulation, compared to nicotine alone, supporting the role of *N*-nitrosamines as promoters of carcinogenesis.

Finally, our data provide clinical evidence that tobacco smoking-related HSCCs produce MMR deficiency that may be related to miRNA deregulation, supporting our current in vivo findings and previous in vitro data [17]. Specifically, our current clinical observations show that the MMR mechanism in HSCC tumors from smokers can be significantly damaged, compared to unaffected ANTs of the same patient, providing evidence of *MSH2* and *hMLH1* downregulation in upper aerodigestive tract tumorigenesis (Figure 8). In addition, similarly to our in vivo findings, data from miRNA analysis in the same human specimens had previously documented a significant deregulation of miR-21, miR-155, and miR-451a. Here, we also show a linear correlation between the transcriptional levels of *hMSH2* and miR-21 expression, supporting the role of miR-21 in regulating MSH2 transcription by *N*-Nitrosamines, in line with our prior in vitro findings (Figure 7) [17]. Finally, both our current in vivo experimental and clinical data show an inverse correlation between hMLH1 and tumor suppressor miR-451a expression, encouraging further exploration of the interactions of miR-451a and hMLH1 in carcinogenesis of the upper aerodigestive tract.

## 5. Conclusions

Our study provides the first insights that NNK-NDEA, combined with or without nicotine, produces downregulation of the *MSH2* and *MLH1* MMR genes, likely via miRNA dysregulation in dysplastic hypopharyngeal mucosa, and further supports the role of NF-κB in TS-associated MMR dysfunction and carcinogenesis in the upper aerodigestive tract. Our data encourage future studies with longer exposure or variable concentrations, to further elucidate a possible in vivo dose-dependent effect of individual or combined *N*-Nitrosamines, NNK and/or NDEA, and nicotine, on the MMR mechanism. In addition, we provide clinical evidence of a dysregulated MMR mechanism, particularly of *hMLH1,* in HSCC tumors of smokers, compared to their ANT. Although further extensive longitudinal studies are needed to investigate the role of miRNAs in regulating MMR genes and provide detailed key molecular checkpoints, the data presented here may also encourage clinical testing for MLH1 and MSH2 that can enhance prognosis and risk assessment of TS-related upper aerodigestive tract malignancies.

## Figures and Tables

**Table 1:** Experimental and control groups of wt-C57BL/6J.

Experimental and Control Groups	Nicotine (NIC) (0.02 μmol/L)	*N-*Nitrosamines [NNK (0.2 mmol/L) + NDEA (0.004 mmol/L)]	2% Saccharin in Water
NIC	*	-	*
NNK-NDEA	-	*	*
NNK-NDEA-NIC	*	*	*
Treated control	-	-	*
Untreated control	-	-	-

-/*, component of the experimental or control fluid.

**Table 2:** Chronic exposure to TS components causes a decrease of MLH1 and MSH2 in premalignant murine hypopharyngeal mucosa.

Target Protein	* NIC	* NNK-NDEA	* NNK-NDEA-NIC
^a^ ***MSH2***	−1.4	−1.6	−2.1
^b^ ***MLH1***	−1.4	−1.5	−1.7

^a^ MutS Homolog 2; ^b^ MutL protein homolog 1; * Relative expression in NIC, NNK-NDEA or NNK-NDEA-NIC experimental groups, versus treated controls.

**Table 3:** MMR mRNA and * miRNA phenotypes associated with human HSCC from smokers.

		† Relative Expression (HSCC/ANT)
**MMR genes**	***hMSH2***	**−48**
	***hMLH1***	**−3**
**miRNA** **markers**	**miR-21**	**28**
	**miR-155**	**23**
	**miR-451a**	**−80**

* miRNA phenotype had previously been identified in these specimens [37]; HSCC, hypopharyngeal squamous cell carcinoma; ANT, adjacent normal tissue; † statistically significant changes in HSCC compared to their ANT.

## Data Availability

The data presented in this study are available in this article (and supplementary material).

## Supplementary Materials

The following supporting information can be downloaded at: https://www.mdpi.com/article/10.3390/curroncol29080437/s1, Supplementary data: Figure S1: Precancerous lesions induced by chronic exposure of murine HM of C57Bl6J to TS components; Figure S2: Enhanced NF-κB activation in dysplastic murine HM exposed to N-nitrosamines plus nicotine compared to normal treated-control HM; Supplementary Table S1: Pack year tobacco smoking history of patients with HSCC; Table S2: Mouse and human genes (targets and reference control genes) GeneGlobe ID and their and detected transcripts, analyzed by real time qPCR; Table S3: Mouse mature miRNAs (targets) and reference RNU6-2 small RNA control, analyzed by real time qPCR, in murine HM; Table S4: Survival data, total C57Bl/6J mice analyzed, and prevalence of those with premalignant lesions of the hypopharyngeal epithelium under its chronic exposure to tobacco smoke components; Table S5: Transcriptional levels of MMR genes (A) in tobacco smoke components exposed murine HM and (B) in human HSCCs and their ANTs from tobacco smokers; Supplementary Table S6: miRNA levels in tobacco smoke exposed murine HM; Supplementary Table S7: Relative expression of hMSH2 and hMLH1 mRNAs and miRNAs in human HSCC compared to their ANT.

## Abbreviations

MMR: mismatch repair; TS, tobacco smoke; HM, hypopharyngeal mucosa; NNK, 4-(methylnitrosamino)-1-(3-pyridyl)-1-butanone; NDEA, N-Nitrosodiethylamine; NIC, nicotine; HSCC, hypopharyngeal squamous cell carcinoma; ANT, adjacent normal tissue.
