# Supplementary material for: The Effect of Tobacco Smoke N-Nitrosamines, NNK and NDEA, and Nicotine, on DNA Mismatch Repair Mechanism and miRNA Markers, in Hypopharyngeal Squamous Cell Carcinoma: An In Vivo Model and Clinical Evidence
Source: Curr Oncol. 2022 Aug 4;29(8):5531–49. doi: 10.3390/curroncol29080437 (PMC9406897; doi:10.3390/curroncol29080437)
Supplement: Supplementary file 1 [file curroncol-29-00437-s001.zip › curroncol-1800645-supplementary.pdf]

Supplementary Materials

The Effect of Tobacco Smoke *N*-Nitrosamines, NNK and NDEA, and Nicotine, on DNA Mismatch Repair Mechanism and miRNA Markers, in Hypopharyngeal Squamous Cell Carcinoma: An In Vivo Model and Clinical Evidence

Sotirios G. Doukas [1,2,3], Dimitra P. Vageli [1,*], Panagiotis G. Doukas [1], Dragana Nikitovic [4], Aristidis Tsatsakis [2] and Benjamin L. Judson [1]

[1] The Yale Larynx laboratory, Department of Surgery, Yale School of Medicine, New Haven, CT 06510, USA; medp2011896@med.uoc.gr (S.G.D.); panagiotis.doukas@yale.edu (P.G.D.); benjamin.judson@yale.edu (B.L.J.)

[2] Department of Forensic Sciences and Laboratory of Toxicology, Medical School, University of Crete, 71003 Heraklion, Greece; tsatsaka@uoc.gr (A.T.)

[3] Department of Medicine, Rutgers/Saint Peter’s University Hospital, New Brunswick, NJ 08901, USA

[4] Department of Histology & Embryology, Medical School, University of Crete, 71003 Heraklion, Greece; nikitovic@uoc.gr (D.N.)

* Correspondence: dimitra.vangeli@yale.edu; Tel.: +1-203-737-1447

Supplementary Table S1. Pack-year tobacco smoking history of patients with HSCC.

Case No. (#)	*Tobacco smoking (py*/ppd^)*
#8	30 py /1 ppd
#4	45 py / 1 ppd
#6	30 py / 1 ppd
#3	40 py / 1 ppd
#2	30 py / 1 ppd
#7	75 py / 1.5 ppd
#1	40 py / 1 ppd
#5	40 py / 2 ppd

*pack-year smoking history, ^ppd: pack of cigarettes per day

Supplementary Table S2: Mouse and human genes (targets and reference control genes) GeneGlobe ID and their detected transcripts, analyzed by real time qPCR, in murine HM and human HSCC and their ANT.

Gene (mouse)	Name	GeneGlobe ID	Detected transcripts	Amplicon length (bp)
Gapdh	Glyceraldehyde-3-phosphate dehydrogenase	QT01658692	NM_008084<br>NM_001289726	144
Msh2	mutS homolog 2	QT00109963	NM_008628	69
Mlh1	mutL homolog 1	QT00138446	NM_026810<br>XM_006511947<br>XM_006511948<br>XM_006511949	133
**Gene (human)**	**Name**	**GeneGlobe ID**	**Detected transcripts**	**Amplicon length (bp)**
hGAPDH	Glyceraldehyde-3-phosphate dehydrogenase	QT00079247	NM_001256799,<br>NM_002046,<br>NM_001289745,<br>NM_001289746	95
Hs_MSH2	mutS homolog 2	QT02564660	NM_000251,<br>XM_005264332	86
Hs_MLH1	mutL homolog 1	QT00028833	NM_000249,<br>NM_001167618-9,<br>NM_001258271-4,<br>XM_005265161,3,4,6	127

Supplementary Table S3: Mouse mature miRNAs (targets) and reference *RNU6-2* small RNA control, analyzed by real time qPCR, in murine HM.

**miRNA (mouse)**	**Target mature miRNA, Sanger Accession)**
miR-21a	mmu-miR-21a-5p, MI0000569
miR-155	mmu-miR-155-5p, MI0000177
miR-34a	mmu-miR-34a-5p, MI0000584
miR-451a	mmu-miR-451a, MI0001730
**Small RNA**	**Control**
RNU6	U6 small nuclear RNA, ENSMUSG00000095132

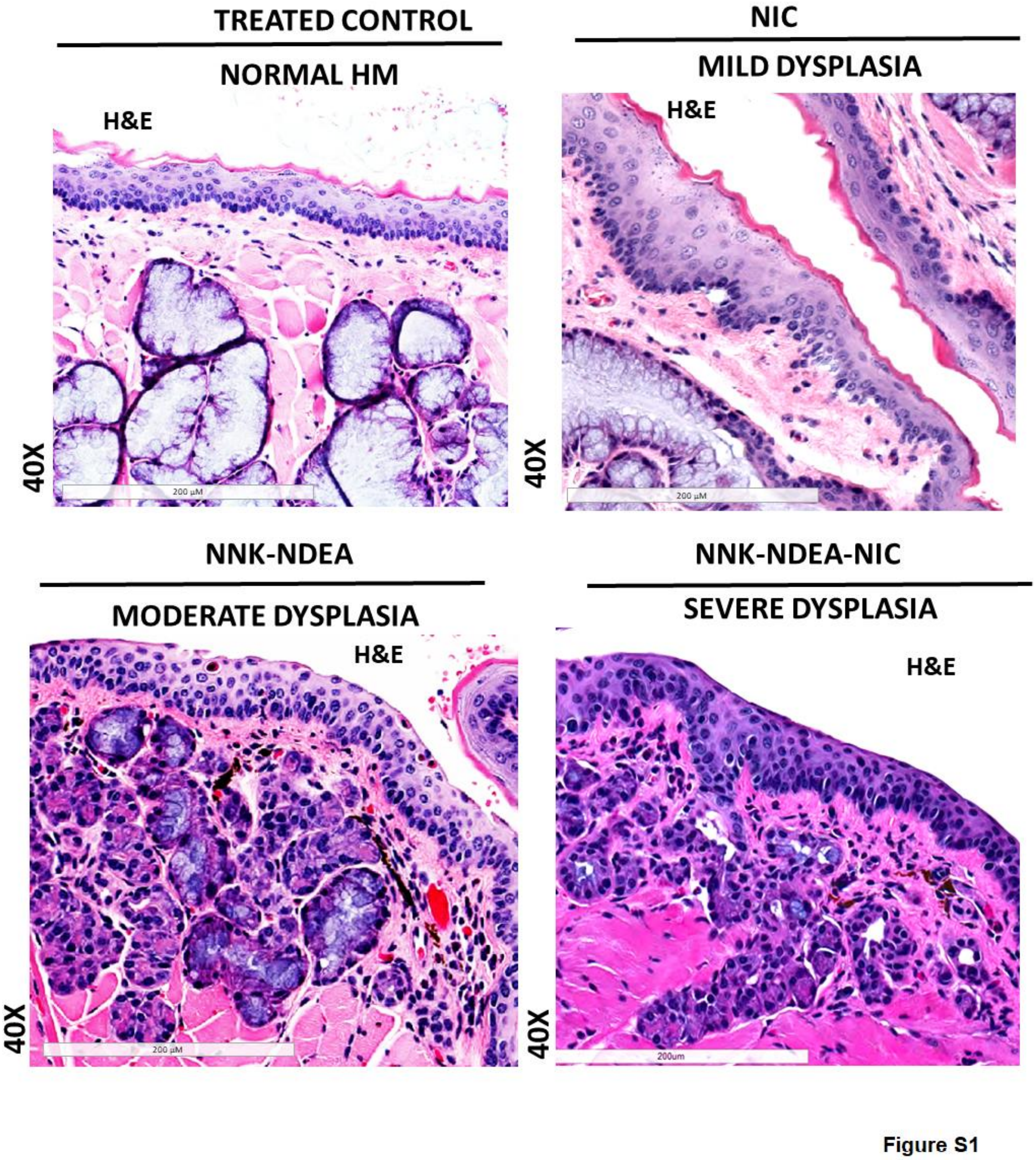

Supplementary Figure S1: Precancerous lesions induced by chronic exposure (14 weeks) of murine hypopharyngeal mucosa (HM) of C57Bl6J to TS components, nicotine (NIC), *N*-Nitrosamines (NNK-NDEA), or their combination (NNK-NDEA-NIC). **A.** Normal control treated-HM. **B.** Mild dysplastic HM (NIC): **C. & D.** Moderate to severe dysplastic HM (NNK-NDEA or NNK-NDEA-NIC). (Hematoxylin & eosin (H&E) staining).

Supplementary Table S4. Survival data, total C57Bl/6J mice analyzed, and prevalence of those with premalignant lesions of the hypopharyngeal epithelium under its chronic exposure to tobacco smoke components, nicotine (NIC), *N*-Nitrosamines (NNK-NDEA), or their combination (NNK-NDEA-NIC).

	Control	NIC	NNK-NDEA	NNK-NDEA-NIC
C57Bl/6J Mice<br>% (survived/total)	100%<br>(8/8)	100%<br>(8/8)	100%<br>(8/8)	100%<br>(8/8)
Total analyzed<br>% (survived/total)	100%<br>(4/4)	100%<br>(4/4)	100%<br>(4/4)	100%<br>(4/4)
Hyperplasia/Dysplasia/<br>% (observed/survived)	0%<br>(0/4)	100%<br>(4/4)	100%<br>(4/4)	100%<br>(4/4)

Supplementary Table S5.

A. Transcriptional levels of MMR genes, *Msh2* and *Mlh1* in tobacco smoke components exposed murine hypopharyngeal mucosa (HM).

**Target gene/ *Gapdh** (*ΔΔ*^{CT})**	**[a]Control**	**[b]NIC**	**[b]NNK-NDEA**	**[b]NNK-NDEA-NIC**
Msh2	7.84E-03	7.62E-03	5.10E-03	4.75E-04
Mlh1	1.23E-02	5.43E-03	6.78E-05	1.83E-05

* Normalization of mRNA levels using *Gapdh*; [a]2% saccharin in drinking water; [b]NIC, NNK-NDEA or NNK-NDEA-NIC solution of 2% saccharin in drinking water.

B. Transcriptional levels of MMR genes, *hMSH2* and *hMLH1* in human HSCCs and their ANTs from tobacco smokers.

**Target gene/ *Gapdh** (*ΔΔ*^{CT})**	**HSCCs**	**ANTs**
hMSH2	4.48E-03	2.14E-01
hMLH1	2.14E-01	6.53E-01

* Normalization of mRNA levels using *hGAPDH*.

Supplementary Table S6: miRNA levels in tobacco smoke exposed murine HM.

	Control	NIC	NNK-NDEA	NNK-NDEA-NIC
miR-21	2.02E+00	4.47E+01	5.27E+01	1.06E+02
miR-155	3.40E-01	3.90E-01	5.50E-01	1.17E+00
miR-34a	2.79E+00	1.10E-01	1.00E-01	9.00E-02
miR-451a	3.60E+00	4.80E-01	5.00E-01	4.00E-01

*Normalization of miRNA levels using small RNA [snRNA RNU6B (RNU6-2)]

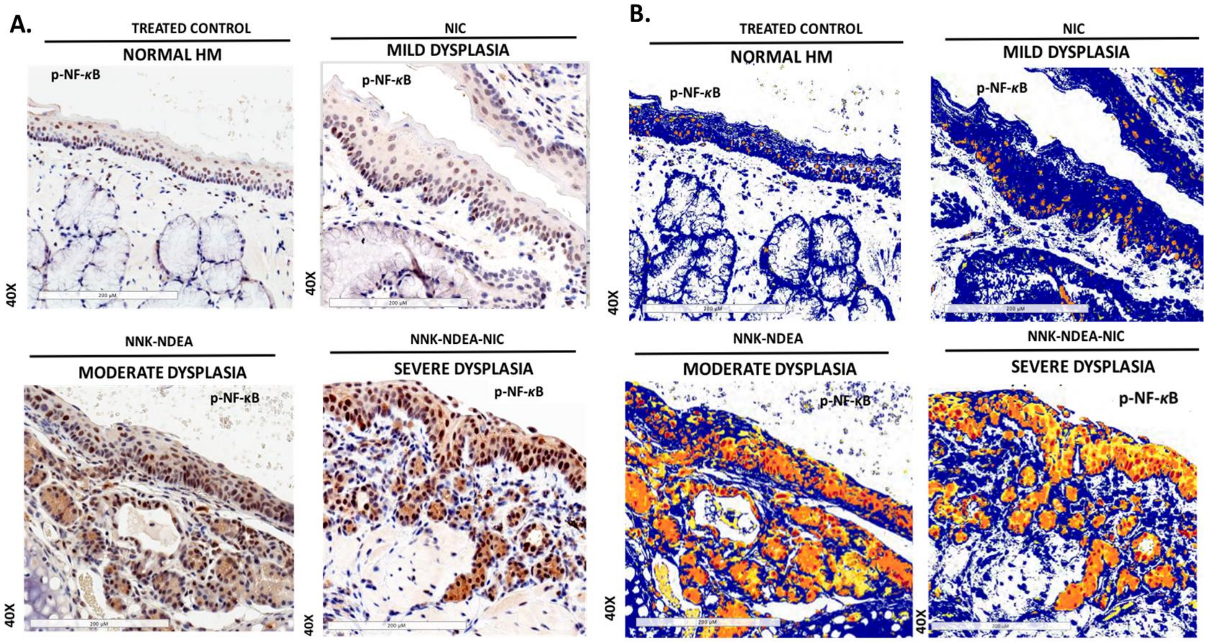

Figure S2

Supplementary Figure S2: Enhanced NF-κB activation in dysplastic murine HM exposed to *N*-nitrosamines plus nicotine compared to normal treated-control HM. A. IHC analysis (brown staining) and **B.** image analysis algorithm(s) for p-NF-κB (p65 S536) of murine hypopharyngeal mucosa (HM) of C57Bl6J mice after 14 weeks of exposure to TS components, nicotine (NIC), *N*-Nitrosamines (NNK-NDEA), or their combination (NNK-NDEA-NIC) and treated control. **B.** Images were captured using Aperio CS2 and analyzed using Image Scope software (Leica Microsystems). In the image analysis algorithm (s), red indicates strong positive nuclear staining of MSH2, orange indicates intense positive cytoplasmic staining of MSH2, yellow indicates weak cytoplasmic staining of MSH2, and blue indicates negative MSH2 staining. *Treated control* (normal HM): sporadic cytoplasmic staining in few basal/parabasal or suprabasal cells. *NIC-treated HM* (mild dysplastic): sporadic cytoplasmic staining in basal/parabasal or suprabasal cells. *NNK-NDEA* and *NNK-NDEA-NIC-treated HM* (moderate-severe dysplastic): intense nuclear and cytoplasmic staining throughout the thickness of dysplastic epithelium.

Supplementary Table S7. Relative expression of *hMSH2* and *hMLH1* mRNAs and miRNAs in human HSCC compared to their ANT.

	MMR genes (†mRNA changes)		‡miRNA expression changes			
Case No. (#)	*hMSH2*	*hMLH1*	miR-21	miR-155	miR-451a	miR-34
#8	234	-1.3	11.9	3	-2.8	1.6
#4	6.6	-3	2.7	9.9	1.4	1.5
#6	191	-8	3.3	5.6	-5.5	1.3
#3	-30	-10	4.1	1.3	2.2	-10.6
#2	1.1	-56	7.1	-4.7	1.7	-65.5
#7	-13	-69	2.6	9.9	-20	1.1
#1	-1355	-76	-2.7	-3.8	-39	3.6
#5	-6.8	-167	-1.2	2.2	-16.7	-1.7

HSCC, hypopharyngeal squamous cell carcinoma; ANTs, adjacent normal tissue; miRNA, microRNA; mRNA, messenger RNA. †mRNA expression changes of MMR genes in HSCC compared to theirs ANTs; ‡miRNA expression changes previously found in HSCC versus controls (37).
